# Supplementary material for: Accurate machine learning model for human embryo morphokinetic stage detection
Source: J Assist Reprod Genet. 2025 Aug 20;42(11):3655–65. doi: 10.1007/s10815-025-03585-4 (PMC12640390; doi:10.1007/s10815-025-03585-4)
Supplement: Supplementary file 1 — (PDF 6.78 MB) [file 10815_2025_3585_MOESM1_ESM.pdf]

Supplementary Figures

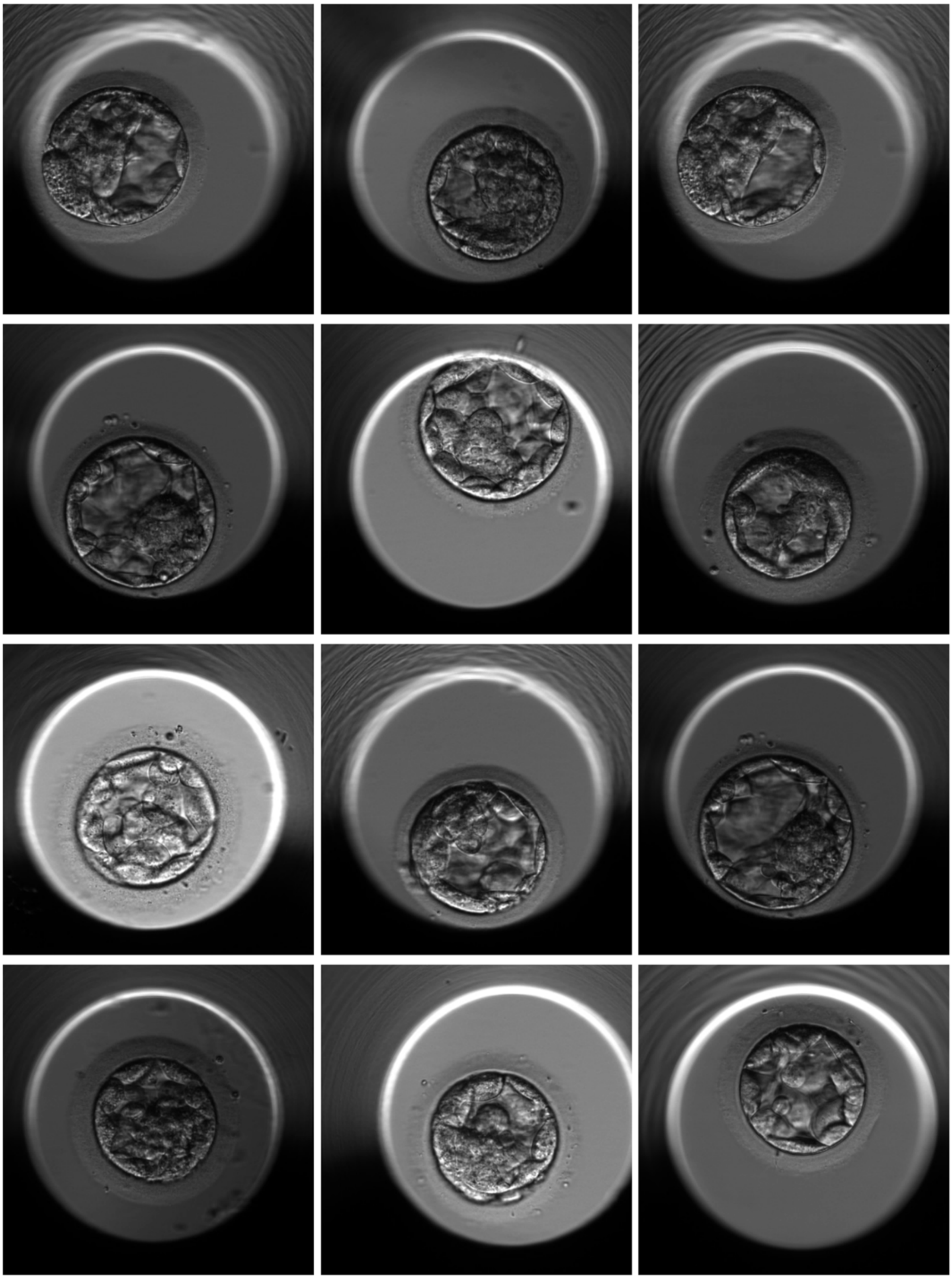

Supplementary Figure 1 A random sample of Images was predicted as tEB by the network but was labelled as tB in the dataset. The misprediction between tEB and tB is among the worst classes for model 2, with 12% of the tB labelled images in the test set predicted as tEB.

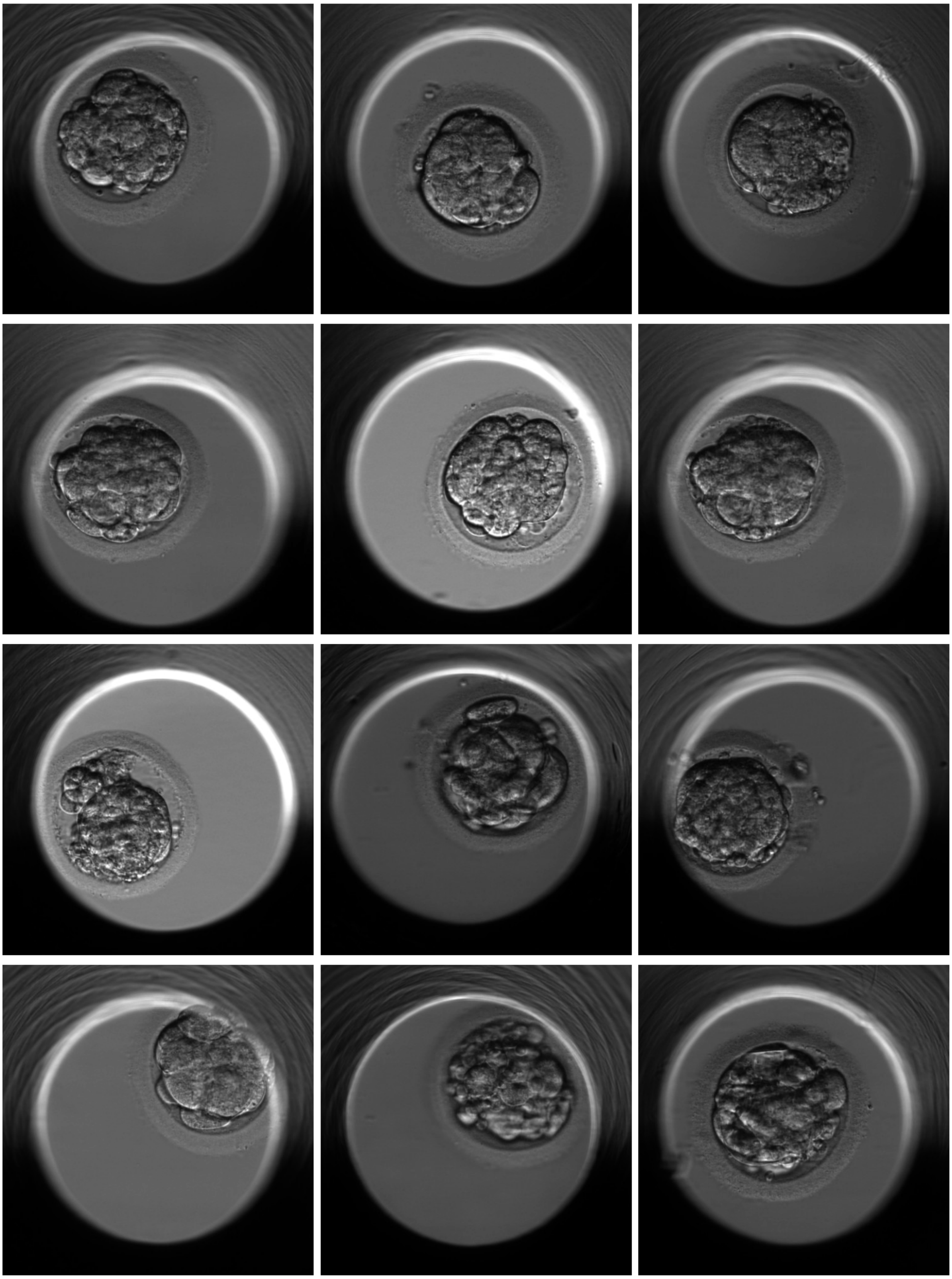

Supplementary Figure 2 A random sample of Images that was predicted as tM by the network but was labelled as tSB in the dataset. The misprediction between tM and tSB is among the worst classes for model 2, with 12% of the tSB labelled images in the test set predicted as tEB.

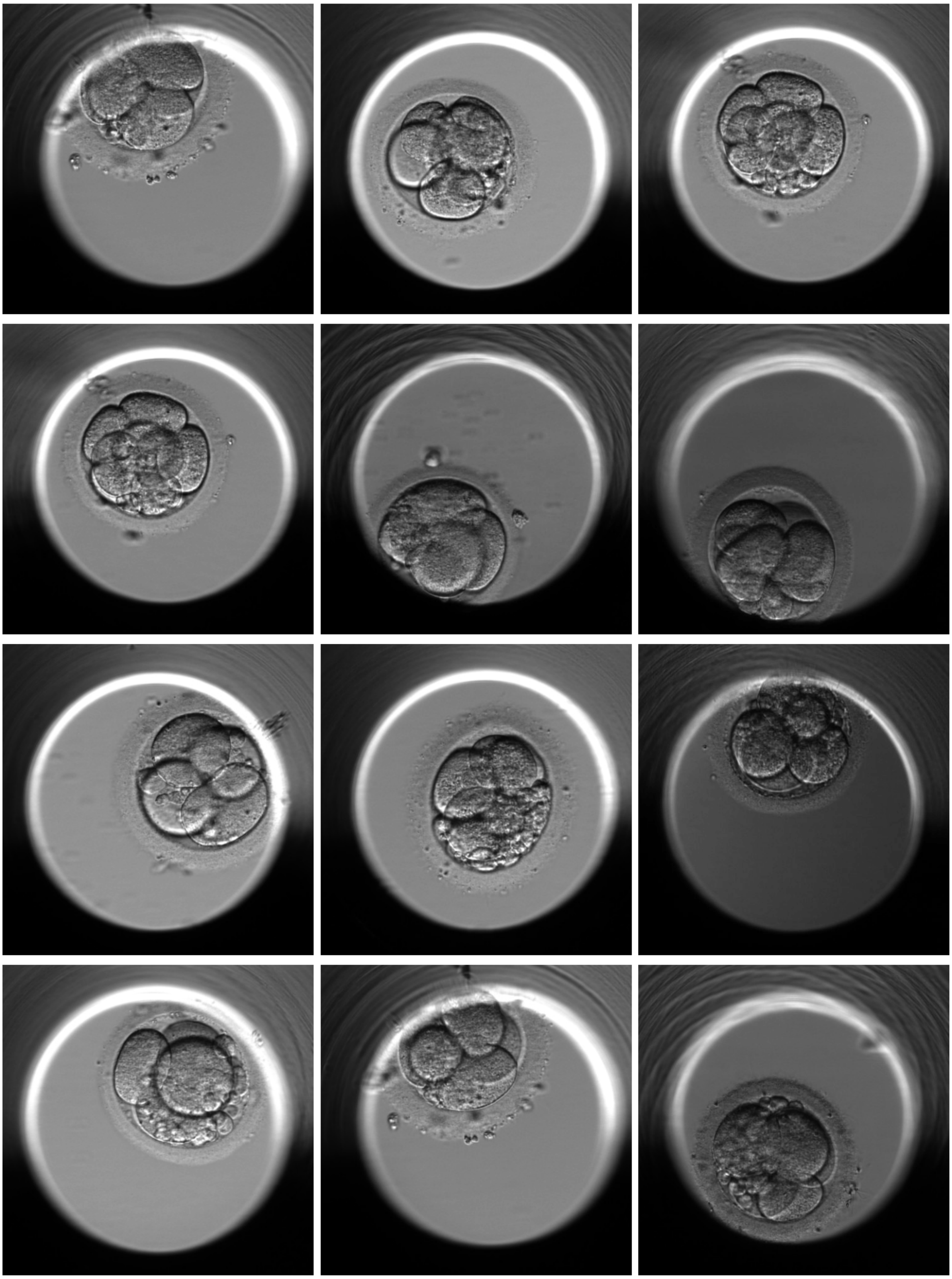

Supplementary Figure 3 A random sample of Images that was predicted as t4 by the network but was labelled as t5 in the dataset. The misprediction between t4 and t5 is among the worst classes for model 2, with 16% of the t5 labelled images in the test set being predicted as t4.

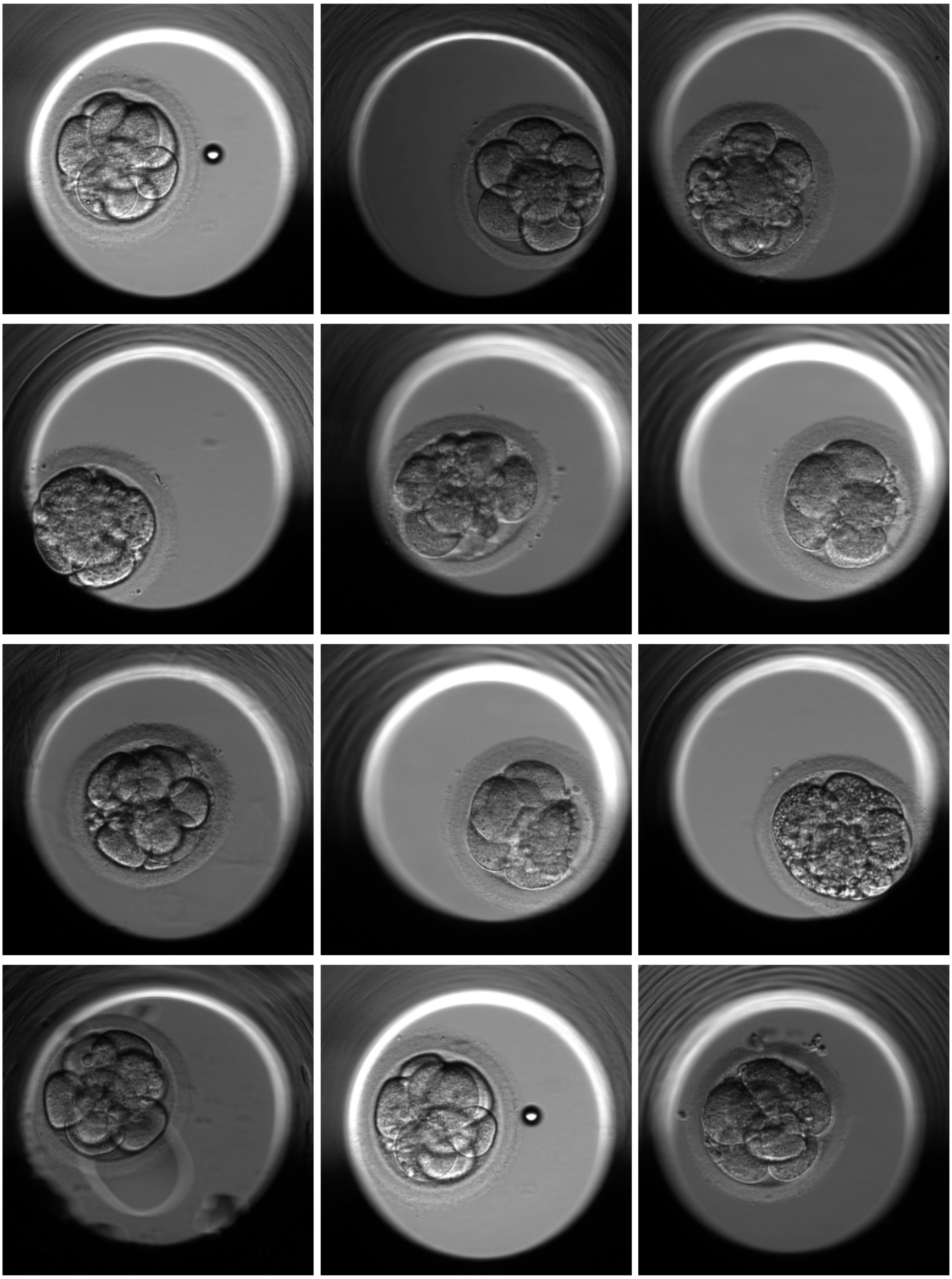

Supplementary Figure 4 A random sample of Images that was predicted as t8 by the network but was labelled as t7 in the dataset. The misprediction between t8 and t7 is among the worst classes for model 2, with 13% of the t7 labelled images in the test set being predicted as t8.

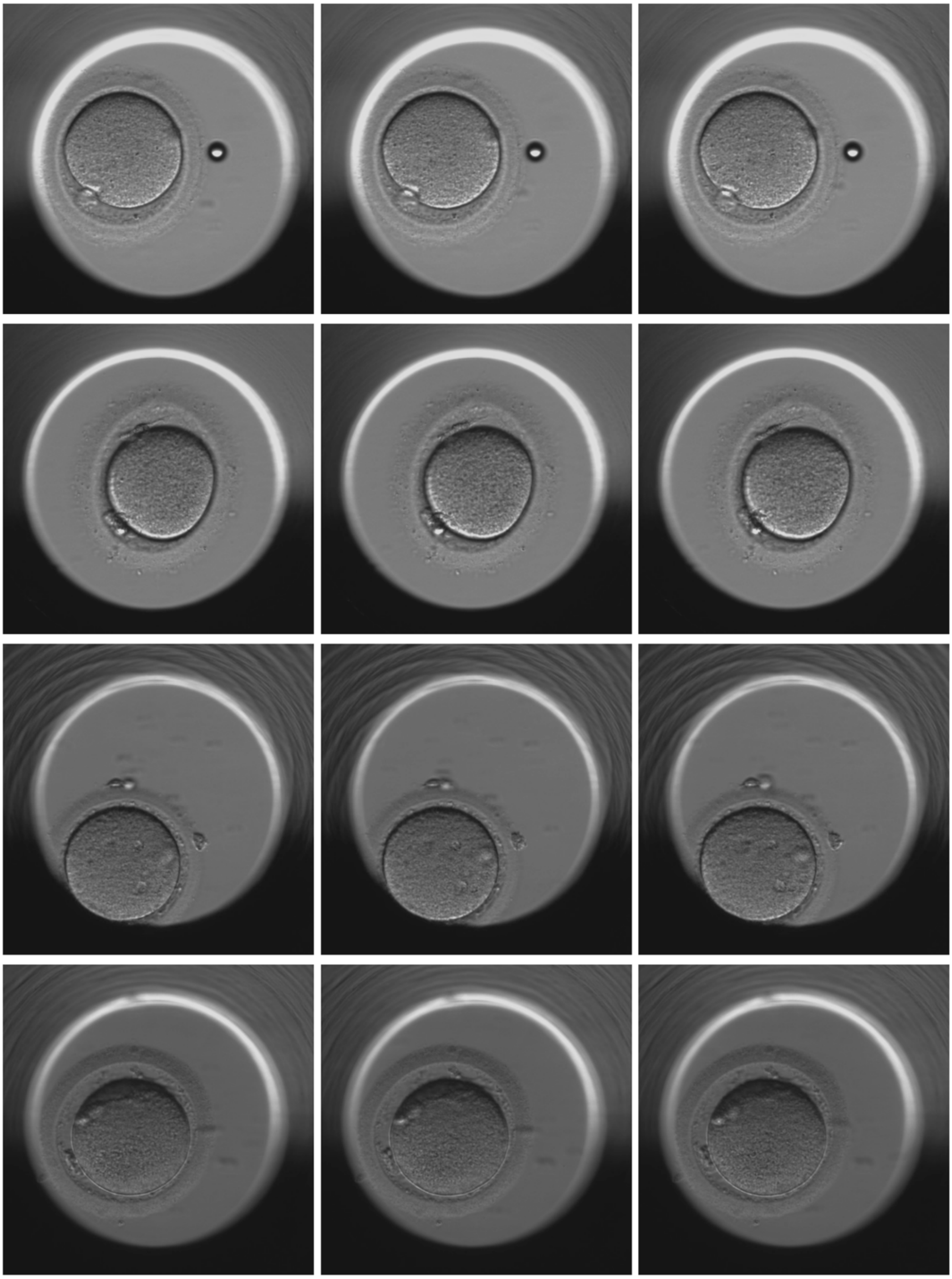

Supplementary Figure 5 Examples from the dataset for embryos classified as tPNa. Each row corresponds to one embryo, and the middle image in each row corresponds to the exact frame reported in the dataset for the morphokinetic event changes. the image on the left is one frame before, and the image on the right is one frame after the labelled change. For this specific morphokinetic event, the pronuclei should be visible in the middle frame and after that.

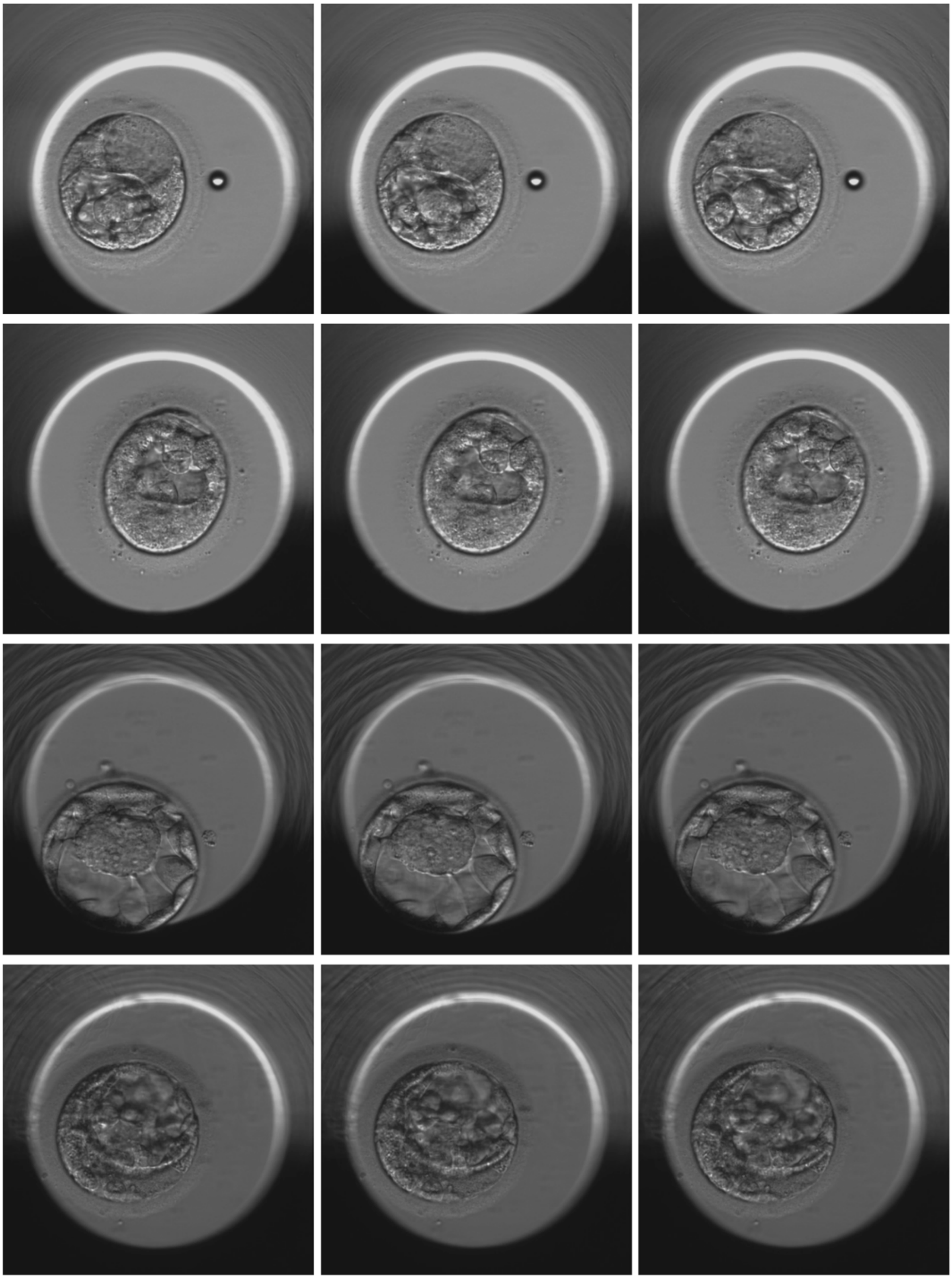

Supplementary Figure 6 Examples from the dataset for class tEB. Each row corresponds to one embryo, and the middle image in each row corresponds to the exact frame reported in the dataset for the morphokinetic event changes. the image on the left is one frame before, and the image on the right is one frame after the labelled change. This shows the subjectivity in the classes between tB and tEB. It is unclear what the criteria for the difference between the classes is.
